# Sarcoidosis Mimicking Primary Lung Cancer on ^99m^Tc-3PRGD2 Scintigraphy in a PTC Patient

**DOI:** 10.3390/diagnostics12061419

**Published:** 2022-06-08

**Authors:** Ye Yang, Xi Jia, Yuanbo Wang, Yan Liu, Yu Liu, Rui Gao

**Affiliations:** 1 Department of Nuclear Medicine, The First Affiliated Hospital of Xi’an Jiaotong University, Xi’an 710061, China; yangye132465798@stu.xjtu.edu.cn (Y.Y.); mydjx@126.com (X.J.); mars8727@xjtufh.edu.cn (Y.W.); liuyan0219@126.com (Y.L.); 2 Department of Pathology, The First Affiliated Hospital of Xi’an Jiaotong University, Xi’an 710061, China; liuyu5208@163.com

**Keywords:** ^99m^Tc-3PRGD2 SPECT/CT, hyper vascularization, sarcoidosis

## Abstract

Sarcoidosis is a multi-system disease of unknown etiology that typically occurs in middle-aged adults, often presenting as the formation of granulomas in various organs, including the lungs. Non-typical pulmonary sarcoidosis is rare, and it isnecessary to distinguish its imaging features from lung cancer and tuberculosis. They may appear as an irregular mass with multiple nodules on thoracic computed tomography (CT). In this case, primary lung cancer was suspected in a 57-year-old papillary thyroid carcinoma patient, as the pulmonary lesions were non-radioiodine avid and progressed shortly afterward. The asymmetrical focal uptake that was demonstrated in integrin receptor imaging (^99m^Tc-PEG4-E[PEG4-c(RGDfK)]2 (^99m^Tc-3PRGD2)) warranted flexible-bronchoscope biopsy. Meanwhile, no evidence of malignancy was found, and pathological manifestations led to the subsequent six months of anti-tuberculosis treatment. Combined with the fact that standard anti-tuberculosis showed no improvement, and the patient’s condition was stabilized by corticosteroid treatment alone, a final diagnosis of sarcoidosis was made by an MDT (multidisciplinary consultation). Reported herein is the first case of a hyper vascularization condition within the non-typical asymmetrical sarcoidosis lesions, which should help to establish that the uptake of 3PRDG2 in sarcoidosis can avoid imaging pitfalls.

**Figure 1:**
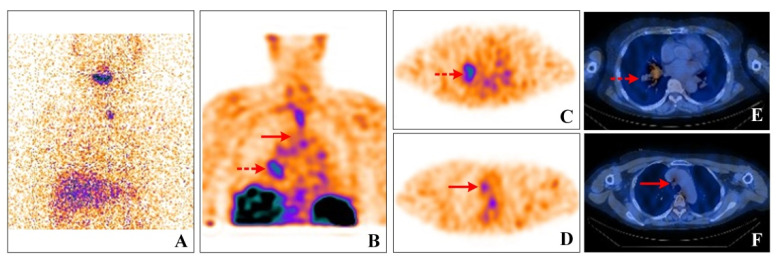
^131^I whole-body scan(WBS) (**A**), ^99m^Tc-3PRGD2 SPECT/CT WBS (**B**), ^99m^Tc-3PRGD2 SPECT/CT imaging (**C**–**F**). A 57-year-old female with papillary thyroid carcinoma (PTC) was suspected of pulmonary metastasis, as multiple pulmonary nodules and enlarged mediastinal lymph nodes (LNs) were found during the preoperative work-up. The tests on post-surgery found undetectable stimulated thyroglobulin levels. Four weeks post-surgery, 150 mCi radioactive iodine therapy (RAI) was administered. Except for faint uptake within the thyroid bed, no concentration in the pulmonary lesions was found (Figure 1A). Six months after RAI, thoracic CT demonstrated apparent progression of the pulmonary lesions. An irregular mass in right hilar with multiple small nodules was detected. Tuberculosis (TB) or primary lung cancer was suspected. At the same time, all the lab tests, including the T-SPOT, TB sputum smear, TB-DNA, and serum tumor markers (CEA, NSE, and SCC), showed negative results. As integrin αvβ3 receptor imaging has advantages in evaluating hyper vascularization in pulmonary lesions [1,2], our center conducted a clinical trial of RGD imaging concerning thyroid cancer and related malignancies. Given that the traditional imaging features may overlap with other pathologic entities in the lung, an integrin αvβ3 receptor imaging ^99m^Tc-3PRGD2 SPECT/CT was performed. Significant focal uptake of ^99m^Tc-3PRGD2 was found in the hilar mass (Figure 1B, dotted arrows; Figure 1C,E), as well as in the enlarged lymph nodes in the neck and mediastinum regions (Figure 1B, dashed arrows; Figure 1D,F). Primary lung cancer was suspected, but flexible bronchoscope biopsy showed non-caseating epithelioid cell granuloma nodules in the lesion.

**Figure 2:**
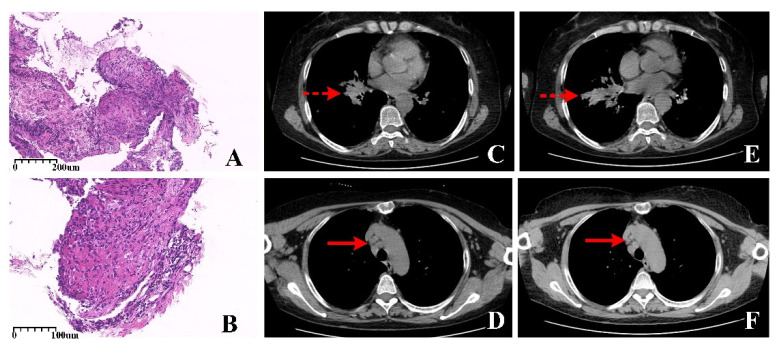
Histopathology (**A**, HE × 100; **B**, HE × 200), axial view of the high-resolution (HR) CT (**C**,**D**, pre-antituberculosis; **E**,**F**, post-antituberculosis). The bronchoscope detected obvious local occupying at the opening of basal segment bronchus of the lower lobe; the lesion was so protruding that the bronchoscope was unable to move forward. Therefore, the samples were taken at the lesion occupying of bronchial orifice. Histological analysis of a specimen showed non-caseating epithelioid granulomas nodules were composed of tightly clustered epithelioid histiocytes and revealed chronic inflammation associated with exuberant granulation tissue, obviously accompanying a large number of multinucleated giant cells and acellular hyaline substances between the nodules (Figure 2A, HE × 100). Additionally, other specimens showed chronic inflammation associated with exuberant granulation tissue and numerous epithelioid cells (Figure 2B, HE × 200). After that, standard anti-tuberculosis treatment was carried out. After six months, significant progression was revealed in the hilar mass (dotted arrows) and the enlarged lymph nodes (dash arrows) on HR CT (Figure 2C,D pre-anti tuberculosis; Figure 2E,F post-anti tuberculosis). Repeated flexible bronchoscope biopsy demonstrated pathological findings consistent with the prior specimens. Therefore, treatment with 0.5 mg/kg prednisolone was started, and all physical findings remained stable due to the corticosteroid treatment alone. A careful review of the clinical history and exclusion of other causes of pulmonary lesions helped make the final diagnosis of sarcoidosis. Sarcoidosis is a systemic disease characterized by a non-caseous necrotizing granuloma, affecting every organ system in the body [3]. A total of 90% of sarcoidosis patients have varying degrees of chest invasion with non-typical presentation in conventional imaging [4]. Additionally, the differential diagnosis mainly includes infections, especially tuberculosis, and malignancies, especially lung cancer and lymphoma. External pulmonary sarcoidosis, as well as symmetrically distributed along the bilateral perihilar, and mediastinal with increased metabolic activity, are the most frequently reported features in ^18^F-FDG, ^68^Ga-DOTATOC, and ^68^Ga-PSMA PET/CT [5,6,7]. However, no significant uptake of the substrate amino acid transporter was observed in sarcoidosis [8]. Different imaging results revealed the heterogeneity and diversity of sarcoidosis. Although those make more challenges for diagnosis, different molecular imaging offers opportunities for effective follow-up treatment when conventional treatment fails. On the one hand, asymmetric nodular changes on metabolic imaging also could be represented in sarcoidosis due to the different metabolic degrees of the lesions, which provided a case for future metabolic imaging to reduce misdiagnosis. On the other hand, the integrin αvβ3 receptor is overexpressed in thyroid cancer cells and neovascular endothelial cells and binds specifically to Argi-nine-glycine-aspartic acid (RGD)-peptide. Thus, ^99m^Tc-3PRGD2 SPECT/CT has long been adopted in evaluating angiogenesis in studies [9,10]. Excellent diagnostic efficacy has been shown to assess metastasis in thyroid cancer in our center [11]. Integrin αvβ3 plays an essential role not only in tumor progression and is also expressed in macrophages, neutrophils, monocytes, and vascular smooth muscle cells [12], giving rise to 3PRGD2 concentrates in macrophage inflammatory responses. The inter-relationship between those inspires that anti-angiogenic therapy may also be helpful in sarcoidosis. Consequently, molecular imaging offers limited diagnostic value for sarcoidosis. It is worth noting that the rare presence of asymmetrical focal hyper vascularization could not rule out the diagnosis of sarcoidosis when tumor markers and tuberculin tests were negative, and the pathology showed granulomatosis. Recognizing the uptake of 3PRDG2 in sarcoidosis can avoid imaging pitfalls.

## Data Availability

Not applicable.
